# Genetic markers for the resistance of honey bee to Varroa destructor

**DOI:** 10.18699/VJ20.683

**Published:** 2020-12

**Authors:** M.D. Kaskinova, L.R. Gaifullina, E.S. Saltykova, A.V. Poskryakov, A.G. Nikolenko

**Affiliations:** Institute of Biochemistry and Genetics – Subdivision of the Ufa Federal Research Center of the Russian Academy of Sciences, Ufa, Russia; Institute of Biochemistry and Genetics – Subdivision of the Ufa Federal Research Center of the Russian Academy of Sciences, Ufa, Russia; Institute of Biochemistry and Genetics – Subdivision of the Ufa Federal Research Center of the Russian Academy of Sciences, Ufa, Russia; Institute of Biochemistry and Genetics – Subdivision of the Ufa Federal Research Center of the Russian Academy of Sciences, Ufa, Russia; Institute of Biochemistry and Genetics – Subdivision of the Ufa Federal Research Center of the Russian Academy of Sciences, Ufa, Russia

**Keywords:** Apis mellifera, Varroa destructor, Varroa resistance, marker-assisted selection, Apis mellifera, Varroa destructor, Varroa-резистентность, маркер-опосредованная селекция

## Abstract

In the mid-20th century, the first case of infection of European bees Apis mellifera L. with the ectoparasite mite Varroa destructor was recorded. The original host of this mite is the Asian bee Apis cerana. The mite V. destructor was widespread throughout Europe, North and South America, and Australia remained the only continent free from this parasite. Without acaricide treatment any honeybee colony dies within 1–4 years. The use of synthetic acaricides has not justified itself – they make beekeeping products unsuitable and mites develop resistance to them, which forces the use of even greater concentrations that can be toxic to the bees. Therefore, the only safe measure to combat the mite is the use of biological control methods. One of these methods is the selection of bee colonies with natural mite resistance. In this article we summarize publications devoted to the search for genetic markers associated with resistance to V. destructor. The first part discusses the basic mechanisms of bee resistance (Varroa sensitive hygienic behavior and grooming) and methods for their assessment. The second part focuses on research aimed at searching for loci and candidate genes associated with resistance to varroosis by mapping quantitative traits loci and genome-wide association studies. The third part summarizes studies of the transcriptome profile of Varroa resistant bees. The last part discusses the most likely candidate genes – potential markers for breeding Varroa resistant bees. Resistance to the mite is manifested in a variety of phenotypes and is under polygenic control. The establishing of gene pathways involved in resistance to Varroa will help create a methodological basis for the selection of Varroa resistant honeybee colonies.

## Abstract (ru)

. В середине ХХ в. был зафиксирован первый случай заражения европейских пчел Apis mellifera L. клещом-эктопаразитом Varroa destructor, изначальным хозяином которого является азиатская пчела Apis cerana. Клещ распространился по всей Европе, Северной и Южной Америке, и единственным континентом, свободным от этого паразита, осталась Австралия. Без обработки акарицидами семья медоносной пчелы погибает в течение 1–4 лет. Использование синтетических акарицидов не оправдало себя – они делают непригодными продукты пчеловодства и у клещей возникает к ним резистентность, что заставляет использовать еще большие концентрации препаратов, которые могут быть токсичны для пчел. Единственная безопасная мера борьбы – использование методов биологического контроля. Одним из таких методов является селекция семей пчел, обладающих естественной резистентностью к клещу. В обзоре обобщены публикации, посвященные поиску генетических маркеров, ассоциированных с устойчивостью к V. destructor. Рассматриваются основные механизмы устойчивости пчел к клещу (Varroa-чувствительное гигиеническое поведение и груминг) и методы их оценки. Обсуждаются исследования, направленные на поиск локусов и генов-кандидатов, ассоциированных с устойчивостью к варроатозу, при помощи картирования локусов количественных признаков и полногеномного поиска ассоциаций. Обобщены исследования транскриптомного профиля Varroa-устойчивых пчел. Рассмотрены наиболее вероятные гены-кандидаты – потенциальные маркеры для селекции Varroa-резистентных пчел. Резистентность к клещу проявляется в виде разнообразных фенотипов и находится под полигенным контролем. Установление генных путей, задействованных в механизме резистентности к Varroa, поможет создать методологическую базу для селекции устойчивых к варроатозу семей A. mellifera.

## Introduction

The Varroa destructor Anderson & Trueman, 2000 is the most widespread and most harmful pest of bees (Anderson, Trueman, 2000; Martin et al., 2012). Review articles devoted to V. destructor deal with various aspects of its biology (Calderon et al., 2010; Rosenkranz et al., 2010; Nazzi et al., 2016; Evans, Cook, 2018), ways to mite control (Chandler et al., 2001; Dietemann et al., 2012; Kamler et al., 2016; Plettner et al., 2017), issues of bee resistance to mite and hygienic behavior (Zakar et al., 2014; Kurze et al., 2016; Locke, 2016a; Leclercq et al., 2017).

The invasion of Varroa has become a challenge for the European bee, since it has not developed the natural defense mechanisms that well developed in the original host of the mite – the Asian bee Apis cerana. The resistance of the Asian bee to the mite is due to the fact that it has well-developed behavioral defense mechanisms and the mite parasitizes mainly on drone brood (Pritchard, 2016). The currently known methods of fighting V. destructor are based on the use of synthetic acaricides and biological control methods (Dietemann et al., 2012; Kamler et al., 2016; Plettner et al., 2017). The problem of acaricides accumulation in beekeeping products and the development of acaricides resistance in the mite make beekeepers refuse to use them. Therefore, biological control methods are of great importance, one of which is the selection of bees that have resistance toward the Varroa mites.

The purpose of this review is to summarize the materials of experimental studies devoted to the establishment of the genetic basis of honey bee resistance to the V. destructor.

## Mechanisms of resistance to varroatosis

There are two main phenotypes associated with resistance to mite: Varroa sensitive hygiene behavior and grooming, which includes auto-grooming (self-cleaning) and allogrooming (cleaning the body of another member of colony).

Before considering the concept of Varroa sensitive hygienic behavior, let’s get acquainted with such a mechanism of protecting bees from brood diseases as hygienic behavior. In 1964 the brood removal behavior of bees infected with American foulbrood was described (Rothenbuhler, 1964). This behavior, called hygienic, consisted of the following actions – detecting, uncapping and removing the infected brood. About twenty years later, Gilliam et al. (1983) showed that hygienic behavior is also effective against ascospherosis. In 1993, the breeding program for honey bee colonies with a high level of hygienic behavior has been started in the University of Minnesota (Spivak, 1996). It was found that hygienic behavior is performed by 15–17 days old bees (Arathi et al., 2003). Bees remove fifth instar larvae infected with the bacterium Paenibacillus larvae (caused American foulbrood) and the fungus Ascosphaera apis (causative agent of ascospherosis) before the pathogens reach the sporulation stage (Spivak, Reuter, 2001; Albo et al., 2017).

In 1997, the Suppression of Mite Reproduction (SMR) phenomenon was described: bee colonies with this phenotype have a low number of reproductively successful female mites (Harbo, Harris, 1999). It soon became clear that SMR is a consequence of specific hygienic behavior aimed at removing a mite, which has offspring. It is known that the foundress mite, after penetrating into an unsealed cell with a bee larva, begins to lay eggs only 3 days after the cell is sealed (Spivak, 1996; Harbo, Harris, 2005; Harris, 2007; Harris et al., 2010; Rosenkranz et al., 2010). The detection and removal of the cells content with mite offspring leads to a reduction in the total number of mites in the bee colony. This type of behavior has been termed Varroa sensitive hygiene (VSH) (Harbo, Harris, 2005).

To assess hygienic behavior, two tests have been developed and are widely used, – freeze-killed brood assay, FKB (Spivak, 1996; Facchini et al., 2019) and pin-killed brood assay, PKB (Gramacho et al., 1999). These tests are often used in experimental studies to analyze resistance toward the Varroa mite, so we will consider a short protocol for their implementation. The brood combs are frozen (FKB) or killed with a pin (PKB) and introduced into the test colony for 24 hours. If colony removes more than 95 % of the killed brood it is considered highly hygienic. VSH assessment is more complex: a section of combs with sealed brood infested with mites is introduced into the test colony and after a week the percentage of uncapped and cleaned cells and other indicators are calculated (Villa et al., 2009). FKBassay was developed to assess hygiene behavior, however Danka et al. (2013) reported that colonies bred for VSH remove frozen brood faster (in 6–12 hours) than colonies bred for FKB assay. At the same time, colonies selected using FKB assay do not cope with the test developed to assess VSH phenotype. Therefore, FKB assay can be used to test VSH phenotype, but this fact requires additional verification.

Grooming behavior is another natural defense mechanism of bees, which consists in the ability of bees to clean themselves (auto-grooming) or other bees (allogrooming) from external parasites and pollution (Boecking, Spivak, 1999; Land, Seeley, 2004). It is strongly expressed in A. cerana (Fries et al., 1996). This is especially true for allogrooming: if an Asian bee cannot remove a mite by itself, it performs a special dance that provokes other bees to perform allogrooming (Land, Seeley, 2004). There are also a difference in grooming between A. mellifera subspecies. For example, Africanized bees remove mites more intensively than European subspecies (Invernizzi et al., 2015). Colonies are assessed for this feature both at the individual (Aumeier, 2001) and colony level (Bienefeld, 1999).

In addition, populations of A. mellifera were identified that survived and coexist with V. destructor for a long time. Evaluation of such colonies showed that they have a high level of Varroa sensitive and grooming behavior (Locke, 2016b). On the basis of genomic and transcriptome studies, loci and genes associated with Varroa resistance were identified.

## Mapping of loci and genes associated with Varroa resistance

Oxley et al. (2010) identified the Hyg1 locus on chromosome 2 associated with hygienic behavior. The 95 % confidence interval of this locus (see Darvasi, Soller, 1997) included genes associated with behavior, smell, development and functioning of neurons, receptor and transcriptional activity. Harpur et al. (2019) based on genome-wide sequencing of drones from two apiaries selected for hygienic behavior and one non-selected apiary identified 73 candidate genes. 49 of them were located near previously identified loci (Oxley et al., 2010; Tsuruda et al., 2012). Of great interest are the abscam, goosecoid (Hoxgene) and tropomysin-2-like genes on chromosome 6, the ortholog of the Drosophila dyschronic gene (GB45054) on chromosome 11, and the insulin-like receptor (GB53353) on chromosome 9. Abscam is known to play an important role in axonal guidance, in particular of olfactory neurons. The goosecoid and tropomysin-2-like genes are also essential for the development of the nervous system. The GB45054 gene is involved in biological processes such as sensory perception of sounds and light stimuli. GB53353 is involved in protein phosphorylation and the transmembrane receptor protein tyrosine kinase signaling pathway. Kim et al. (2019) performed genome-wide sequencing of A. m. caucasica with high hygienic behavior and A. m. carnica with a low level of hygiene. They obtained 20 SNP markers associated with hygienic behavior, and candidate genes were identified for three of them. SNP1 is located in the twitchin (chromosome 2), in the previously identified locus Hyg1 (Oxley et al., 2010). SNP8 and SNP9 are located in the gene encoding a peroxidase-like protein (chromosome 4)

In studies (Oxley et al., 2010; Harpur et al., 2019; Kim et al., 2019) hygiene behavior was assessed using FKB assay, and, as it was said, colonies selected on FKB do not always successfully cope with a mite. However, given that Varroa sensitive and general hygienic behavior are based on the same mechanism (detecting and uncapping diseased brood), results obtained by these authors should not be excluded from further consideration.

Genome-wide analysis of VSH was carried out by research groups from the USA (Tsuruda et al., 2012) and Germany (Spotter et al., 2012, 2016). Tsuruda et al. (2012) identified a locus on chromosome 9 associated with VSH phenotype. This locus contains the NorpA2 gene (homologue of the D. melanogaster NorpA) and the dopamine receptor Dop3. NorpA2, encoding phospholipase C, is associated with learning and memory formation in the honey bee (Suenami et al., 2018). Whereas dopamine plays a critical role in the formation of aversive memory in insects (Beggs, Mercer, 2009)

Spotter et al. (2012) analyzed three samples of bees with different levels of VSH and developed a differentiating panel of 44,000 SNPs. In next study (Spotter et al., 2016) they identified 6 SNPs associated with resistance towards the V. destructor. For four of them, candidate genes were proposed: AdoR, Cdk5alpha, Octbeta2R, and Obp1. The identified SNPs are not located in candidate genes themselves, but are localized near them. Therefore, their role in the formation of VSH phenotype has yet to be proven. The authors substantiated the choice of these candidate genes by their function. Adenosine receptors (encoded by the AdoR gene) belong to the family of G proteincoupled receptors and are involved in extracellular adenosine signaling. Adenosine is an important regulator of the nervous system; it is involved in the modulation of synaptic plasticity (Dolezelova et al., 2007). Cdk5alpha encodes an activator of the cyclin-dependent kinase gene Cdk5. Cdk5 regulates many cellular processes (neuronal migration, axon guidance, ensuring the stability of microtubules and synapses, etc.), and it has been shown that in the Asian bee A. cerana Cdk5, together with its activator gene, is involved in the cell response to oxidative stress (Zhao et al., 2018). The biogenic amine octopamine is an important neurotransmitter, modulator and hormone in invertebrates. It was shown that the octopamine receptor gene Octbeta2R plays an important role in the formation of adaptations in the high-mountain population of A. m. monticola (Wallberg et al., 2017). Obp1, expressed in the antennae of worker bees, is responsible for the perception of queen pheromones (Lartigue et al., 2004), and probably for the perception of other olfactory signals

In addition to the colonies selected for hygiene, there are populations that coexisted with V. destructor for a long time without acaricide treatment (in review Locke, 2016b). These populations have become the object of close scrutiny by geneticists. Behrens et al. (2011) analyzed offspring of two hybrid queens from a Varroa tolerant colony from the Gotland population. They uncapped the sealed drone brood and estimated the number of mites with and without offspring. Colonies with mites without offspring were considered as resistant. Using 488 SSR markers for mapping, they identified a locus on chromosome 7 associated with this phenotype. This locus contains two important candidate genes, orthologs of D. melanogaster genes, foxo (GB11764, a transcription factor in the insulin signaling pathway) and futsch (GB11509, inducessynaptic plasticity in neurons). Lattorff et al. (2015) based on data from Behrens et al. (2011) also analyzed bee colonies from the Gotland population. They compared colonies before (2000) and after (2007) selection using 39 SSR markers on chromosomes 4 and 7. 11 candidate genes were identified on chromosome 7, including 10 protein-coding genes and one gene of long non-coding RNA, the target of which is unknown. The authors propose the oxidoreductase gene GMCOX18 as a promising candidate gene. Oxidoreductases are involved in glucose metabolism and cuticle biosynthesis. Therefore, the authors hypothesized that the GMCOX18 may play a role in altering substances secreted by bee larvae, which are required to trigger oogenesis in a mite.

Among the genetic markers found in Varroa tolerant colonies using SNP mapping (Conlon et al., 2018) candidate genes involved in the synthesis of ecdysone are distinguished. It is known that V. destructor cannot synthesize ecdysone itself and receives it from bees. Ecdysone is necessary for mite to activate the reproductive cycle, while in insects it initiates molting and metamorphosis. Conlon et al. (2018) performed genome-wide sequencing of drones from Varroa tolerant colonies from Sweden and identified a locus on chromosome 15 associated with tolerance to the mite. This locus includes three genes involved in ecdysone synthesis: Mblk-1, Cyp18a11 and Phantom. They continued their research by performing genome-wide sequencing of drones from another Varroa tolerant population, the Toulouse population from France (Conlon et al., 2019). As a result, 9 SNPs associated with Varroa olerance were identified, and three of them were located in the transcription factor Mblk-1

A search was also carried out for genes associated with the grooming behavior of bees. Arechavaleta-Velasco et al. (2012) identified a locus on chromosome 5 and named it “groom-1”. It includes 27 candidate genes, three of which (Atlastin, Ataxin, AmNrx1) are associated with the development of the nervous system and behavior.

## Transcriptome analysis of Varroa resistance

After the decoding of the honeybee’s genome, studies of its transcriptome were initiated. Differential gene expression analysis is often used to find candidate genes. It allows finding out how the activity of certain genes can affect the mechanisms of resistance.

A comparative analysis of the transcriptome profile of colonies with high and low levels of hygiene behavior (Boutin et al., 2015) revealed 28 genes with increased expression in the former. Most of them were located at previously identified loci (Oxley et al., 2010; Spotter et al., 2012; Tsuruda et al., 2012). Of great interest as markers are genes of cytochrome P450 gene superfamily (Cyp4AZ1, Cyp4g11, Cyp6AS11, Cyp6AS8), which are over-expressed in non-hygienic bees. Cytochrome P450 enzymes degrade odorant and pheromone molecules (Feyereisen, 1999), thereby reducing the ability of bees to detect infected brood.

Transcriptomic analysis of colonies with VSH phenotype was performed by two groups (Le Conte et al., 2011; Mondet et al., 2015). Le Conte et al. (2011) identified 39 differentially expressed transcripts in the brains of bees with VSH phenotype compared to control bees without VSH. Among the genes with increased expression in the brain of VSH bees, the authors emphasize PRL-1, which encodes tyrosine phosphatase, and GB16747. It was later shown that the expression of the GB16747, involved in the metabolism of ascorbate/ aldarate, increases in response to infection with V. destructor (McDonnell et al., 2013). The Cyp4g11 and Obp3 genes and three exons of the Dscam were under-expressed.

Mondet et al. (2015) found 258 differentially expressed transcripts in the antennae of worker bees with and without VSH phenotype. Among genes involved in redox metabolism 12 genes were over-expressed and 3 genes were underexpressed in bees with VSH. Four genes that control the immune response, in particular the Def1 and Def2, were under-expressed. Of particular interest are genes associated with olfaction (Obp3, Trh, OR85b-like, CSP2, NT-7, Obp14, et al.). Proteomic studies have also shown the involvement of the Obp genes (Obp17 and Obp18) in the formation of VSH phenotype (Hu et al., 2016). Differential expression of the Obp genes indicates that the olfaction plays an important role in VSH.

Analysis of two susceptible and two tolerant colonies (Navajas et al., 2008) showed that mite-tolerant bees undergo changes in the expression of genes that regulate the neurons development and sensitivity, as well as the olfaction (orthologs of D. melanogaster genes, which are over-expressed: poe, GluClα, para, Dhc64c, and which are under-expressed: futsch, scratch, fringe, Dscam, etc.). Colonies were used as tolerant if they had not been treated with acaricides for 11 years and had a low level of mite infestation (the authors counted mites at the hive bottom 4 times a year for 5 years). In susceptible colonies the level of mite infestation was 10 times higher.

Jiang et al. (2016), comparing transcriptome profiles of a V. destructor tolerant colony that survived without acaricide treatment for 58 months and a susceptible colony that died from varroatosis within 17 months, identified 6 candidate genes. Of these, 4 encode proteins of cytochrome P450. The Cyp6AS12 and Cyp6BE1 genes were over-expressed in pupae of the tolerant mite-infested colony. Cyp6BE1 and Cyp9Q3 were over-expressed in adults from a tolerant mite-free colony relative to the same mite-infested colony, whereas at the pupal stage there were no significant differences in expression levels of the two genes

Conlon et al. (2019) measured the expression of Mblk-1, Cyp18a11, and Phantom genes in workers and drones larvae from Varroa tolerant colonies to verify the results of genome wide analysis. The expression pattern of genes involved in ecdysone biosynthesis (in particular, the transcription factor Mblk-1) differed in drone larvae and worker larvae. If a mutation occurs in the genes responsible for the ecdysone synthesis, this can lead to a malfunction of the mite development cycle. It is possible that the preference of the drone brood by the mite and its more successful reproduction in it is a consequence of the differences in the level of ecdysone expression in the drone and bee brood.

Transcriptome analysis confirmed the contribution of the neurexin I gene (Arechavaleta-Velasco et al., 2012) to grooming behavior. In colonies with a high level of grooming behavior, the expression of this gene was increased (Hamiduzzaman et al., 2017).

Transcriptomic studies were also performed for the Asian bee A. cerana. Ji et al. (2014) compared the transcriptomes of nurse bees of A. cerana before and after infection with V. destructor (after 24 hours). Among genes whose expression increased in response to mite infection were genes associated with olfaction (Obp4, Obp17, Obp18, Dscam), as well as transcription factors (CREB-like 2-like and Mblk-1). Diao et al. (2018) showed that A. cerana has more immune genes and genes encoding antimicrobial peptides than A. mellifera. However, A. cerana has fewer genes encoding odorant-binding proteins (Obp) and olfactory receptors. This suggests that after the divergence, the European bee lost some of its genes due to the lack of Varroa pressure, and when faced with it, A. mellifera activated other mechanisms. Differences in the methylation levels of genes responsible for learning and memory were also recorded. The formation of long-term memory and synaptic plasticity requires activation of neuronal signaling pathways. Transcriptome analysis showed that, in A. cerana, the expression of genes involved in signaling pathways (cAMP-PKA, MAPK, and CaMK IV) increases in response to mite infection.

## Genetic markers of bee resistance to varroatosis

The above mentioned studies narrowed down the list of potential loci and candidate genes that determine the resistance of bees to the Varroa mite. Each of the studies identified its own candidate genes. Overlaps (coincidences of results) were obtained mainly for those studies in which the same methods for assessing the resistant phenotype were used (see the Table).

**Table 1.**
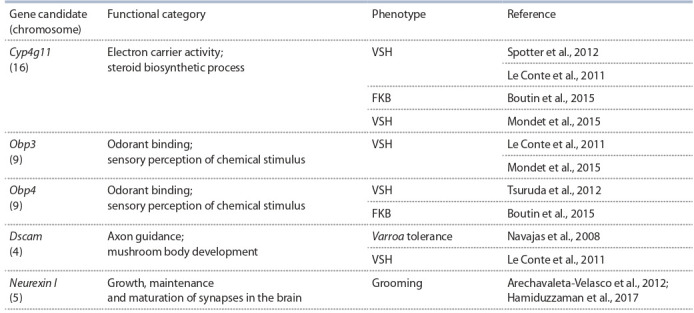
Overlapping candidate genes associated with Varroa destructor resistance

In studies of the transcriptome profile of the brain (Le Conte et al., 2011) and antennae (Mondet et al., 2015) of VSH bees, a common candidate gene Cyp4g11 was identified. In the brain, Cyp4g11 expression was decreased, and in the antennae it was increased. Decreased expression of the Cyp4g11 gene in the brain was also shown for colonies with high hygiene behavior tested with FKB (Boutin et al., 2015). In addition, the Cyp4g11 gene is located at one of the loci previously identified in VSH bees (Spotter et al., 2012). It is currently unknown what function Cyp4g11 performs in the honey bee organism. Cytochrome P450 genes are involved in ecdysteroids metabolism, detoxification of xenobiotics and destruction of odorant molecules (Feyereisen, 1999).

A common candidate gene Obp3 was identified for VSH colonies (in two studies independently). In VSH bees, the expression of this gene is increased in antennas (Mondet et al., 2015), while in the brain it is decreased (Le Conte et al., 2011). For one more gene from the Obp family, an overlap was found: the Obp4, which are under-expressed in the brain of bees selected for FKB (Boutin et al., 2015), is located at one of the loci on chromosome 9, identified earlier (Tsuruda et al., 2012).

The overlap was also shown for Varroa tolerant (Navajas et al., 2008) and VSH (Le Conte et al., 2011) colonies. Dscam expression (GB15141) was under-expressed in Varroa tolerant bees (Navajas et al., 2008). In a study (Le Conte et al., 2011), three exons of the Dscam gene also were under-expressed.

Common candidate genes with the Asian bee A. cerana were also identified. The resistance of the Asian bee to Varroa is the key to understanding the resistance of the European bee. The presence of overlapping genes such as Mblk-1, Dscam, and Obp4 (Ji et al., 2014) confirms this. Further research is needed to establish role of these genes in the mechanism of Varroa resistance in bees

## Conclusion

Genomic and transcriptome studies have shown that genes associated with visual and olfactory perception, development and functioning of the nervous system (learning and memory formation) play the main role in Varroa sensitive hygiene behavior. Receptor genes are of great interest, most of which belong to the family of G protein-coupled receptors (dopamine, adenosine, and octopamine receptors). Some of the identified candidate genes can be successfully used as markers for the selection of specific subspecies or lines of bees for which they were obtained (Haddad et al., 2015; Kim et al., 2019), some of genes needs testing on other populations (Le Conte et al., 2011; Boutin et al., 2015; Mondet et al., 2015; Spotter et al., 2016; Hamiduzzaman et al., 2017). Some candidate genes are associated with a general immune response (Le Conte et al., 2011; Jiang et al., 2016). Further study of some genes (Ji et al., 2014; Lattorff et al., 2015; Conlon et al., 2019), for example, genes for ecdysone biosynthesis, will help to shed light on the nature of the parasite–host relationship, in particular the question of why the mite in the original host reproduces more successfully on the drone brood. Do not forget that the mite is a parasite, and, like many parasites, some of its life support systems are reduced. Finding these pain points of the Varroa mite can also help fight varroatosis.

The resistance of the honey bee to the V. destructor mite is under polygenic control. The European bee was able to use other gene pathways to provide its defense against the V. destructor, despite the short period of time since the mite invasion. Establishing these pathways will help create a methodological basis for breeding Varroa resistant A. mellifera colonies.

## Conflict of interest

The authors declare no conflict of interest.
